# Dose dependent effects of cadmium on tumor angiogenesis

**DOI:** 10.18632/oncotarget.16572

**Published:** 2017-03-25

**Authors:** Tianshu Wei, Jin Jia, Youichiro Wada, Carolyn M. Kapron, Ju Liu

**Affiliations:** ^1^ Medical Research Center, Shandong Provincial Qianfoshan Hospital, Shandong University, Jinan, Shandong, China; ^2^ The Research Center for Advanced Science and Technology, Isotope Science Center, The University of Tokyo, Komaba, Meguro-Ku, Tokyo, Japan; ^3^ Department of Biology, Trent University, Peterborough, Ontario, Canada

**Keywords:** tumor angiogenesis, cadmium, dose dependent effect, endothelial cells, oxidative stress

## Abstract

Angiogenesis is crucial for tumor growth and metastasis. Cadmium (Cd) exposure is associated with elevated cancer risk and mortality. Such association is, at least in part, attributable to Cd-induced tumor angiogenesis. Nevertheless, the reported effects of Cd on tumor angiogenesis appear to be either stimulatory or inhibitory, depending on the concentrations. Ultra-low concentrations of Cd (<0.5 μM) inhibit endothelial nitric oxide synthase activation, leading to reduced endothelial nitric oxide production and attenuated tumor angiogenesis. In contrast, low-lose Cd (1-10 μM) up-regulates vascular endothelial growth factor (VEGF)-mediated tumor angiogenesis by exerting sub-apoptotic levels of oxidative stress on both tumor cells and endothelial cells (ECs). The consequent activation of protein kinase B/Akt, nuclear factor-κB, and mitogen-activated protein kinase signaling cascades mediate the increased secretion of VEGF by tumor cells and the up-regulated VEGF receptor-2 expression in ECs. Furthermore, Cd in high concentrations (>10 μM) induces EC apoptosis via the activation of caspase-3, resulting in destruction of tumor vasculature. In this review, we summarize the current knowledge concerning the roles of Cd in tumor angiogenesis, with a focus on molecular mechanisms underlying the dose dependent effects of Cd on various EC phenotypes.

## INTRODUCTION

Angiogenesis refers to the physiological process in which new blood vessels grow from pre-existing vessels [1, 2]. Driven by pro-angiogenic stimuli, pericytes surrounding the normally quiescent endothelial cells (ECs) detach from the basement membrane [3]. Meanwhile, junctions between ECs dilate, enabling the extravasation of plasma proteins which serve as a provisional framework of the extracellular matrix (ECM) [3]. As a result, ECs migrate, proliferate and form tubes which eventually fuse with neighboring vessels [3–5]. Angiogenesis is vital for normal physiological processes of fetal development and wound healing [3, 6, 7]. However, it is also fundamental for solid tumor growth and metastasis [8]. Tumor angiogenesis provides tumors with oxygen (O_2_) and nutrients, and also aids the disposal of tumor metabolites [7, 9]. Tumor sizes are limited to 2mm in diameter without neovascularization [7]. Moreover, tumor angiogenesis often results in hyperpermeable vasculature with a twisted appearance [10]. Aberrant vasculature in turn aggravates regional hypoxia and acidosis, both of which promote tumor progression [10].

Vascular endothelial growth factor (VEGF) is the predominant mediator of tumor angiogenesis [11]. Malignant cells produce VEGF to promote angiogenesis even before the formation of a visible tumor [8]. Nevertheless, due to the exceptional growth rate of cancerous cells, solid tumors still experience hypoxia despite the already increased vessel formation [8]. Oxygen insufficiency stabilizes hypoxia inducible factor-1α (HIF-1α), a constituent of the transcription factor HIF-1 [11–15]. Activated HIF-1 translocates to the cell nucleus where it binds to promoter regions to promote VEGF expression [16]. VEGF signaling is mediated by VEGF receptor-2 (VEGFR-2) on ECs [11]. Upon binding with VEGF, VEGFR-2 undergoes tyrosine phosphorylation, triggering multiple down-stream signaling events involving activation of mitogen-activated protein kinases (MAPKs) and protein kinase B (PKB)/Akt, which promote EC survival and proliferation [11, 15]. In addition, pro-inflammatory cytokines released by cancerous cells promote EC migration by increasing vascular permeability [3, 17]. Angiogenesis is regulated by a number of stimulators and inhibitors [11]. Inhibitors of angiogenesis have been developed as an attempt to improve cancer treatments [8]. For example, combined therapy with VEGF antagonists and chemotherapy effectively reduced tumor size and invasiveness [10]. On the other hand, environmental chemicals, including toxic metals, may facilitate tumor growth by stimulating angiogenesis [18, 19].

Cadmium (Cd) is a naturally occurring element that can be found both in the atmosphere and in soil [20]. Since the 1940s, Cd has been used widely in industrial processes [20]. Epidemiological evidence suggests that Cd poses a significant health threat because it substantially increases the risk of cancer, renal failure, osteoporosis, and developmental abnormality [20–25]. For non-occupationally exposed populations, Cd exposure normally results from tobacco consumption or ingestion of contaminated substances [21]. Following absorption by either lung or the intestinal epithelium, Cd enters the systemic circulation [26]. Cd exists as a mixture of free cations and metal compounds in blood [26]. Since Cd has a high affinity to thiol groups, plasma proteins containing thiol groups including albumin, metallothionein (MT), and glutathione (GSH) are considered as major carriers of Cd [26, 27]. Both GSH and MT are potent antioxidants, binding of Cd with these proteins neutralizes Cd toxicity [28–30]. Nevertheless, increased level of Cd depletes anti-oxidative enzymes, and thus induces oxidative stress which affects the surrounding cells [26, 31]. Oxidative stress associated with sub-apoptotic dose of Cd activates pro-survival signaling, leading to enhanced cell proliferation and malignant transformation [9, 22, 23, 32–34]. As a result, Cd has been characterized as a Group 1 human carcinogen by the International Agency for Research on Cancer [21].

As Cd directly alters signaling cascades in both tumor cells and ECs, it has been linked with tumor angiogenesis [1, 9, 21, 22, 35–37]. Exposure to Cd increases the production of VEGF by cancerous cells [9, 35]. Cd also directly enhances EC survival and proliferation by up-regulating the expression of VEGFR-2 [38]. Therefore, Cd-induced tumor angiogenesis contributes, at least in part, to the association between high Cd intake and increased cancer mortality [7, 21]. However, Cd has also been described as an inhibitor of angiogenesis [38–45]. The apparent discrepancy between studies calls for an in-depth review regarding the effect of Cd on tumor angiogenesis. This review will provide a detailed analysis of the interactions between Cd and tumor vasculature, and discuss potential mechanisms underlying the dose dependent effect of Cd.

## ULTRA-LOW DOSE CD ATTENUATES ANGIOGENESIS BY INHIBITING ENOS ACTIVITY

Blood Cd concentration serves as a biomarker for Cd exposure level [21]. Data from two Swedish studies indicated that blood Cd concentration in non-occupationally exposed population may range from just above 0 μM to 0.05 μM [21, 46, 47]. Nonetheless, the blood Cd concentration of human varies remarkably subject to age, gender, diet, residential area, and smoking status [21, 47].

Cd in ultra-low concentrations ( < 0.5 μM) attenuates angiogenesis in both the wound healing assay and the chick choriollantoic membrane (CAM) assay [40]. In addition, ultra-low concentrations of Cd reduce bradykinin (BK), a powerful angiogenic agent, and mediate both tube formation in 3D matrigel matrix and *ex vivo* angiogenesis in CAM models [39]. Mechanisms behind these observations have not been fully understood, but such anti-angiogenic effects of Cd might be mediated partially by the blockade of eNOS activity [39, 40]. eNOS is an enzyme in ECs that catalyzes nitric oxide (NO) production [48–50]. Canonically, activation of eNOS is achieved by binding of a calcium/CaM complex to the CaM-binding region of eNOS [50–52]. Interaction with heat shock protein 90 (Hsp90), a chaperone protein, causes membrane-associated eNOS to dissociate from caveolin-1 (cav-1) while undergoing phosphorylation [48, 50, 53]. Phosphorylation of eNOS leads to a flux of electrons through its reductase domain and thus facilitates the oxidative reaction in which L-arginine is transformed to L-citrulline and NO [48, 54–57].

**Figure 1:**
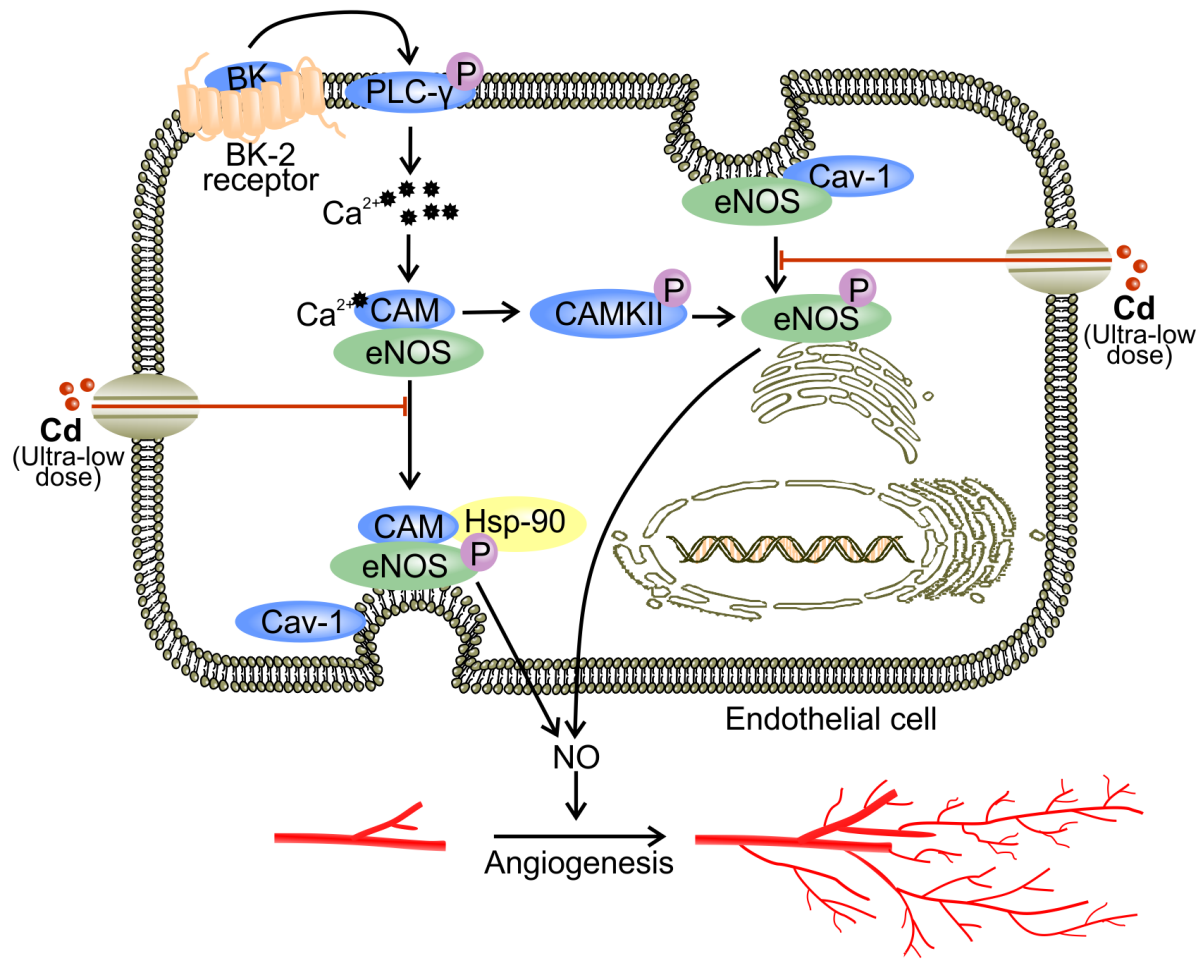
Ultra-low dose of cadmium inhibits angiogenesis by down-regulating eNOS activity At a concentration lower than 0.5 μM, Cd down-regulates BK-induced eNOS activation [39]. Binding between BK and BK2 receptor initiates down-stream signaling of PLC-γ, which involves the up-regulation of intracellular Ca^2+^ levels and activation of CaM [51]. Activated calcium/CaM complex binds to eNOS to trigger its canonical activation involving Hsp90 [48, 50, 53]. In addition, calcium/CaM complex stimulates CaMKII which activates eNOS by direct phosphorylation [51]. Ultra-low dose Cd also impedes eNOS perinuclear translocation [39, 40]. Excessive binding of eNOS to the plasmalemma may lead to cav-1-mediated inhibition of eNOS activity [59].

Upon treatment with ultra-low dose Cd, phosphorylated eNOS in human umbilical vein endothelial cells (HUVECs) is decreased [39, 40]. The reduction in activated eNOS is accompanied by a decrease in NO production [40]. Hence, Cd might directly inhibit eNOS phosphorylation, leading to reduced eNOS activation [40]. Meanwhile, when ECs are treated with ultra-low dose of Cd, BK-induced perinuclear translocation of eNOS is abolished [39]. BK is able to initiate eNOS phosphorylation [39]. Soluble BK binds to the membrane-bound BK2 receptor and activates phospholipase C-γ (PLC-γ), which up-regulates Ca^2+^ levels in the cytoplasm [51]. Elevated cytoplasmic Ca^2+^ levels facilitate the binding between calcium/CaM complex and eNOS [51]. In addition, calcium/CaM complex activates CaM kinase II (CaMKII) which directly phosphorylates eNOS [51, 58]. While membrane association is essential for eNOS activation, restricting eNOS to the caveolae-rich plasmalemma increases the binding between eNOS and cav-1 [48, 59]. Cav-1 binding inhibits the enzymatic activity of eNOS [59]. Therefore, ultra-low dose Cd decreases eNOS signaling *via* the inhibition of eNOS phosphorylation and perinuclear translocation [39] (Figure 1).

Cd also appears to compete with Ca^2+^ for entry into cells [32, 48]. This mechanism potentially explains the decrease in intracellular Ca^2+^ level in Cd treated ECs [39]. Since Ca^2+^ is required for eNOS activation, the competition between Cd^2+^ and Ca^2+^ for passage through ion channels might be another mechanism underlying Cd-reduced NO production [48] (Figure 1). Furthermore, Cd competes with zinc (Zn) for binding sites on proteins [60]. Since myc-associated zinc-finger protein (MAZ) is a promoter of eNOS, the replacement of Zn by Cd in MAZ might attenuate eNOS activity [48, 60]. By suppressing eNOS activation, ultra-low concentrations of Cd reduce NO production by ECs [39, 40]. NO is responsible for regulating vascular tone, EC proliferation, and angiogenesis [50]. NO signaling is orchestrated *via* S-nitrosylation which covalently incorporates NO into a thiol group on the target protein [61]. Under normoxic conditions, S-nitrosylation stabilizes HIF-1α and initiates the transcription of VEGF [16, 61, 62]. NO also contributes to the accumulation of HIF-1α by inhibiting protein hydroxylase domain containing protein 2 (PHD 2) [63, 64]. Hence, decreased NO due to exposure to ultra-low dose Cd reduces VEGF expression. In addition, hypoxia facilitates the binding between cytochrome *c* oxidase and NO [61, 65, 66]. Such binding increases intracellular O_2_ levels by reducing mitochondrial respiration [66]. Combined with NO insufficiency, PHD is activated and promotes the proteasomal degradation of HIF-1α [65, 66]. Therefore, reduced NO level as a result of ultra-low dose Cd exposure leads to decreased VEGF production and impaired angiogenesis [61, 65].

## LOW-DOSE CD STIMULATES ANGIOGENESIS BY UP-REGULATING VEGF EXPRESSION

Low-dose Cd (1 μM-10 μM) promotes tumor angiogenesis in a series of experimental models [9, 35, 38–40, 67]. At this level of exposure, Cd does not induce death of vascular cells [68, 69], however, it might alter the phenotype of both non-endothelial cells and ECs [35, 70]. Low-dose Cd induces oxidative stress, which is characterized by elevated intracellular level of reactive oxygen species (ROS) [9, 29]. By binding to GSH and MT, Cd^2+^ impairs the ability of cells to dispose ROS [32]. In addition, free Cd^2+^ increases ROS formation by damaging the mitochondria and activating NADPH oxidase [32]. Furthermore, Cd may indirectly up-regulate ROS by displacing endogenous Fenton metals, such as Fe^2+^, from proteins [32]. By increasing ROS generation, Cd activates PKB/Akt, NF-κB, and MAPKs, resulting in endothelial cell activation and tumor angiogenesis [9, 38, 67, 71].

### PKB/Akt signaling

**Figure 2:**
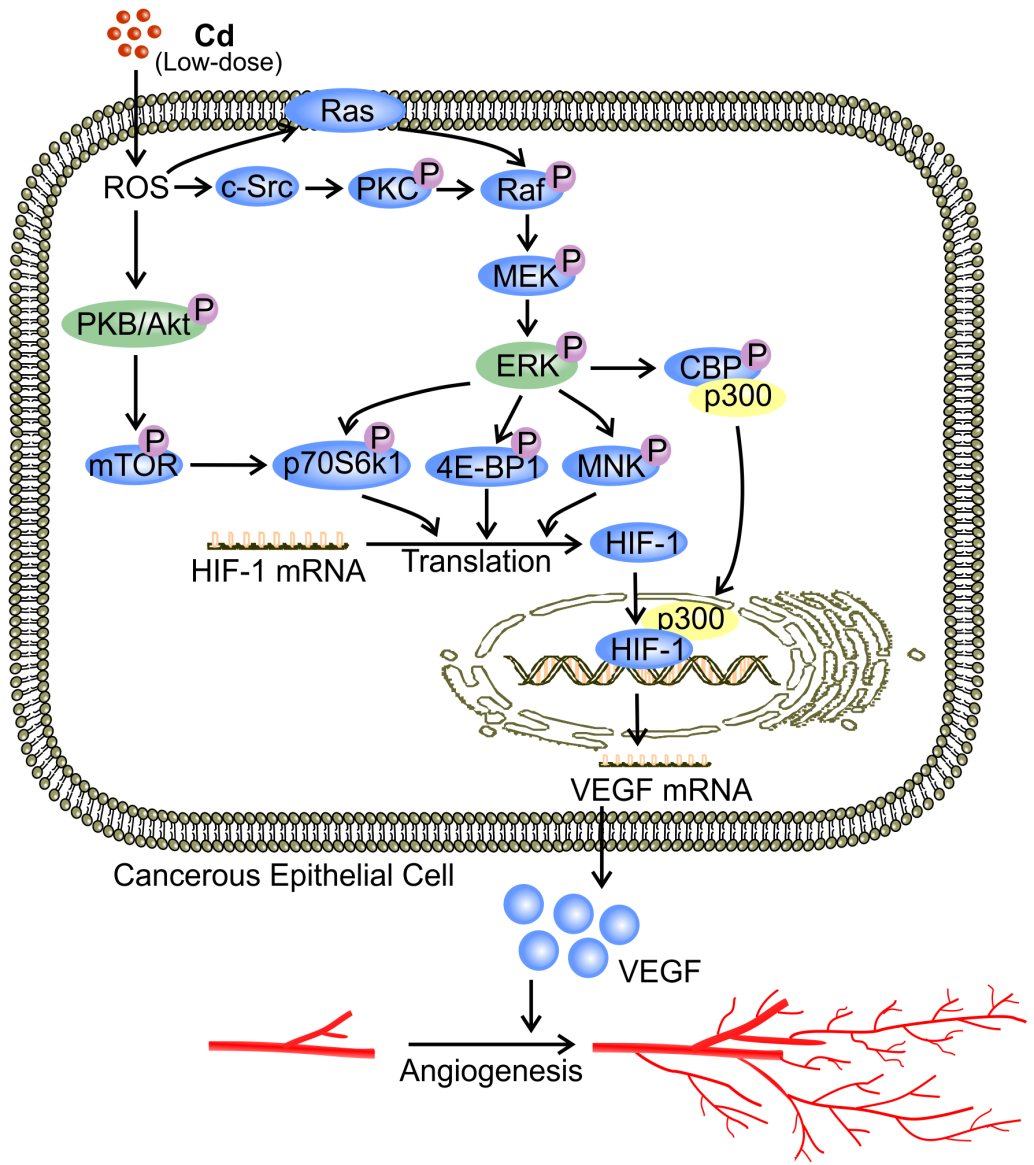
Low-dose Cd induces VEGF expression ***via*** activation of Akt and ERK signaling in cancerous cells. At a concentration between 1 μM and 10 μM, Cd activates PKB/Akt and ERK in cancerous cells by increasing ROS levels [9]. Activation of ERK *via* Raf/MEK is either Ras dependent or through c-Src-activated PKC [95]. PKB/Akt activation phosphorylates down-stream substrates including mTOR and p70S6K1 [9]. Activated ERK phosphorylates 4E-BP1, p70S6K1, and MNK, while triggering dissociation of the CEP/p300 complex, resulting in the binding of p300 with HIF-1 [16, 96]. Together, these signaling events enhance the translation and activity of HIF-1 [16, 72, 96]. Binding between HIF-1 and the promoter region of VEGF up-regulates VEGF production by cancerous cells [16].

PKB, also known as Akt, is a serine/threonine-specific protein kinase invovling in the transcriptional induction of protein expression [72, 73]. PKB/Akt is activated by phosphatidylinositol-4,5-bisphosphate-3-kinase (PI3K) while it controls many downstream components such as mammalian target of rapamycin (mTOR) [72, 73]. PKB/Akt is a primary target molecule in Cd-induced tumor angiogenesis [9]. In non-ECs, Cd (5 μM) triggers activation of PKB/Akt and its down-stream effectors mTOR and p70S6kinase (p70S6K1) [9], which mediates VEGF production [74]. Activated p70S6K1 phosphorylates eukaryotic translation initiation factor 4B (eIF4B), facilitating the recruitment of eIF4B to the pre-initiation complex of protein synthesis [75]. Subsequently, the unwinding of HIF-1 mRNA is promoted along with the induction of HIF-1 translation [16, 72, 75]. Elevated HIF-1 level leads to increased VEGF transcription [9, 16] (Figure 2). In addition, p70S6K1 phosphorylation is associated with actin filament remodeling which promotes cell migration [76]. Accordingly, Cd-conditioned ECs demonstrate enhanced tube formation in a CAM assay [9]. Both enhanced production of VEGF and augmented EC migration induced by PKB/Akt signaling might contribute to low-dose Cd-induced tumor angiogenesis.

**Table 1:** Dose dependent effects of Cd on tumor angiogenesis

Cd conc.	Mechanism of action	Effect on angiogenesis	Ref
<0.5 μM	eNOS inhibition	Inhibitory	39, 40
4 μM	NF-κB activation	Stimulatory	67
5 μM	Akt and ERK activation	Stimulatory	9
5 & 10 μM	Activation of ERK, JNK, and p38 MAPK	Stimulatory	38
>10 μM	p38 MAPK activation	Inhibitory	43
50 μM	Blockage of VEGF signaling and increased Ang-2 expression	Inhibitory	41, 42

### NF-κB signaling

**Figure 3:**
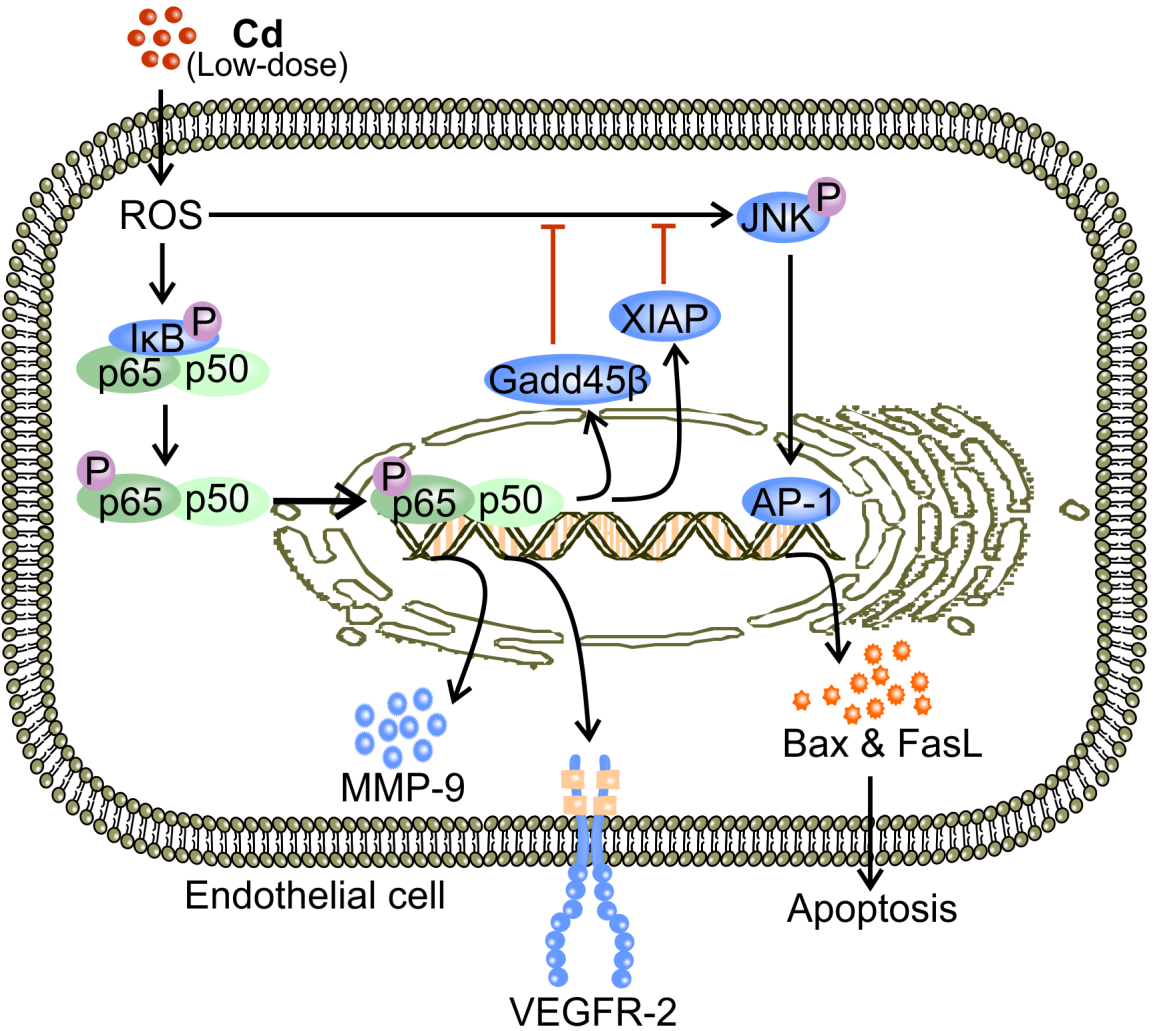
Low-dose Cd activates NF-κB signaling and promotes VEGFR-2 expression while inhibiting JNK-mediated apoptosis in ECs Low-dose Cd (1-10 μM) induces formation of ROS which phosphorylates IκB [67, 77]. Phosphorylation of IκB releases NF-κB (p65/p50 dimer) which then translocates to the nucleus to promote the transcription of VEGFR-2 [71, 77]. Activation of NF-κB leads to expression of proteins that inhibit JNK, including XIAP and Gadd45β [67, 81, 82]. Therefore, Cd-activated NF-κB inhibits JNK-mediated EC apoptosis while promoting VEGFR-2 expression [67].

NF-κB is a transcription factor consisting different combinations of the Rel-family-members such as RelA (p65) and RelB (p50) [67, 77, 78]. It broadly influences gene expression to regulate cell survival, proliferation, and differentiation [77, 78]. Usually, NF-κB dimers reside in the cytosol and are bound to their inhibitory proteins, inhibitor κB (IκB) [77]. Low-dose Cd (4 μM) activates NF-κB by promoting the degradation of IκBα in human renal glomerular endothelial cells [67]. Activated NF-κB dimers then translocate to the cell nucleus where they bind to the promoter regions of pro-angiogenic proteins [67, 77]. NF-κB activation facilitates the expression of VEGFR-2 by ECs [38, 71, 79] (Figure 3). Being the predominant mediator of VEGF signaling, phosphorylated VEGFR-2 activates down-stream pro-angiogenic and pro-survival signaling including the aforementioned PLC-γ and PI3K [80]. Therefore, activation of NF-κB signaling promotes tumor angiogenesis by enriching VEGFR-2 availability.

X-chromosome linked inhibitor of apoptosis (XIAP) and Gadd45β are products of NF-κB activation induced by low-dose Cd [67, 81, 82]. Both proteins have been independently shown to inhibit c-Jun N-terminal kinase (JNK) activation [81, 82]. Stress activated JNK is associated with nuclear translocation of activator protein-1 (AP-1) [83], which promotes the transcriptional expression of Bax and Fas ligand (FasL) [83, 84]. Bax-facilitated release of cytochrome *c* and FasL-engaged Fas mediate apoptosis *via* the intrinsic mitochondrial pathway [84–86]. In the presence of NF-κB inhibitor, low-dose Cd increased JNK phosphorylation, which resulted in a substantial decrease in viable cell count [67, 87]. On the other hand, activated NF-κB reduced the level of phosphorylated JNK and maintained the number of viable ECs [67]. Therefore, by effectively inhibiting JNK-mediated apoptosis, low-dose Cd induced NF-κB activation promotes angiogenesis by maintaining EC survival [67] (Figure 3). In addition, NF-κB activation by exposure to low-dose Cd up-regulates the expression of matrix metalloproteinase-9 (MMP-9) in ECs [88, 89] (Figure 3). MMP-9 promotes the dissociation of EC from the basement membrane, which is essential for the induction of tumor angiogenesis [3, 90]. Genetic ablation of MMP-9 prevents the initiation of tumor angiogenesis in mouse models [91]. The proteolytic activity of MMP-9 also enables it to degrade and remodel the ECM, allowing ECs to migrate [91, 92]. Moreover, ECM degradation by MMP-9 releases VEGF, the potent promoter of tumor angiogenesis [91, 93].

### ERK signaling

ERK1/2 belongs to the MAPK family and has a conventional role in pro-survival signaling [94]. Similar to its effect on PKB/Akt, low-dose Cd (5 μM) increased ERK phosphorylation in human lung epithelial cells by inducing ROS formation [9]. ROS is a well-established activator of ERK mediated by the classic Ras-Raf-MEK pathway [95]. Activation of Raf may also be achieved through c-Src-activated protein kinase C (PKC) [95]. Activated ERK phosphorylates eukaryotic translation initiation factor 4E-binding protein (4E-BP1), p70S6K1, and MAP kinase interacting kinase (MNK) [9, 16, 96]. These signaling events result in increased HIF-1α mRNA translation [16]. Consequently, HIF-1α-mediated VEGF production is increased [9]. ERK is also involved in the transcriptional activation of HIF-1 [16]. ERK phosphorylates CBP/p300 and thus increases HIF-1α/p300 complex formation [16]. Enhanced HIF-1α translation and activation eventually up-regulate VEGF expression [9] (Figure 2).

Low-dose Cd also activated ERK signaling in ECs with an increase in VEGFR-2 expression [38]. Meanwhile, inhibitors of ERK signaling reduces the level of VEGFR-2 [38]. Therefore, Cd-activated ERK up-regulates VEGFR-2 in ECs [38]. The resulting enhanced VEGF signaling contributes, at least in part, to the Cd-induced increase in EC proliferation [80, 97]. Specifically, VEGF-A has been found to activate an orphan nuclear receptor transcription factor TR3 in HUVECs [80, 98]. TR3 then mediates the expression of cell cycle genes which promote EC proliferation [80, 98]. By stimulating EC proliferation, Cd-enhanced activation of ERK signaling in ECs facilitates tumor angiogenesis [1].

### JNK signaling

**Figure 4:**
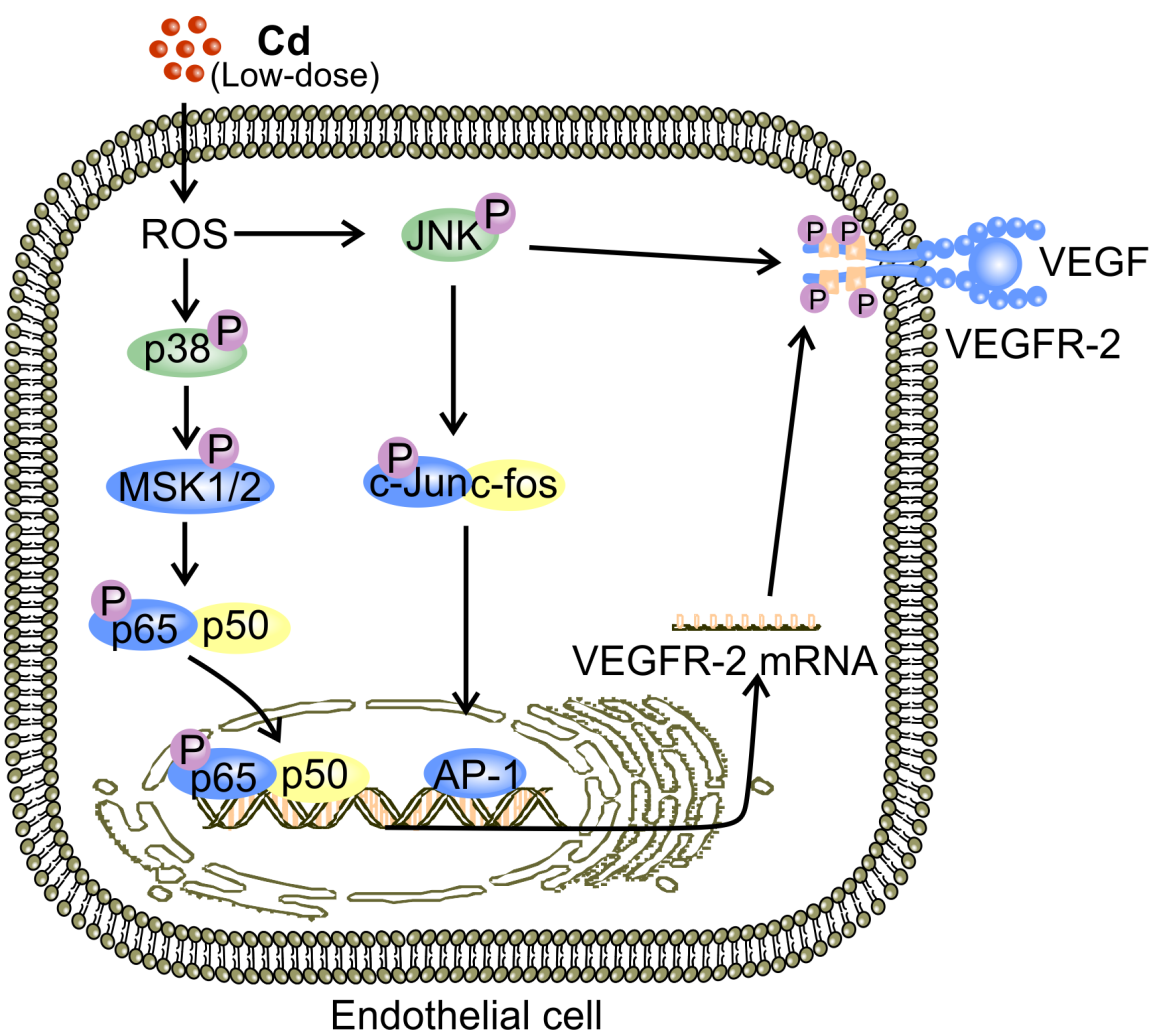
Putative mechanisms for low-dose Cd-activated JNK and p38 MAPK to facilitate expression and activation of VEGFR-2 in ECs JNK-mediated elevation in VEGFR-2 expression is observed in ECs [38]. Activation of JNK by low-dose (1-10 μM) Cd-induced ROS triggers nuclear translocation of AP-1 [83, 84, 102]. Binding of AP-1 to the relevant promoters initiates transcriptional expression of a plethora of proteins including VEGFR-2 [38, 84]. ROS-induced p38 MAPK activation also accounts for elevated VEGFR-2 expression in ECs exposed to low-dose Cd possibly *via* MSK1/2 [94]. Eventually, NF-κB activation leads to up-regulation of VEGFR-2 [71].

JNK is a MAPK that mediates both pro-apoptotic and pro-survival signaling [94, 99, 100]. JNK is phosphorylated under stresses, such as elevated ROS levels [101]. Binding of c-Jun and c-fos (AP-1 complex), the downstream targets of JNK activation, to the promoter regions of DNA initiates transcriptional production of respective genes [83, 84, 102]. Upon exposure to low dose Cd, JNK phosphorylation was increased in a dose dependent manner, peaking at 10 μM, and the expression levels of both VEGF and VEGFR-2 were elevated with an increase in cell viability [38]. Inhibition of JNK activation substantially decreased VEGFR-2 expression in HUVECs [38]. JNK also mediates sustained VEGFR-2 phosphorylation, which is essential for the transduction of VEGF signaling [103] (Figure 4). Furthermore, inhibitor of JNK reduces the release of VEGF by human coronary smooth muscle cells [104]. Hence, JNK activation is essential for VEGF production by non-endothelial cells [104]. Therefore, in addition to maintaining VEGFR-2 phosphorylation, JNK activation by low-dose Cd exposure might promote tumor angiogenesis by up-regulating both VEGF and VEGFR-2 [38, 103, 104].

### p38 MAPK signaling

Similar to JNK, p38 MAPK is also a mediator of both pro-survival and pro-apoptotic signaling [105, 106]. Meanwhile, it is required for VEGF-induced EC migration [106]. Inhibition of p38 MAPK signaling also resulted in reduced VEGFR-2 expression in HUVECs [38]. Nonetheless, the pro-angiogenic activity of p38 MAPK remains to be comprehensively characterized. A potential explanation for p38 MAPK-mediated increase in VEGFR-2 expression in ECs is the ability of p38 MAPK to activate NF-kB by activating mitogen- and stress-activated kinases (MSK1/2) [38, 71, 94] (Figure 4). Importantly, it appears that members of the MAPK family, ERK, JNK, and p38 MAPK, produce a plethora of down-stream signaling events under the stimulation of low-dose Cd. The overall effect of these is enhanced VEGF signaling and tumor angiogenesis [38]. However, interactions between these signaling events remain to be fully understood.

## HIGH-DOSE CD IMPAIRS ANGIOGENESIS BY REDUCING VIABLE ECs

**Figure 5:**
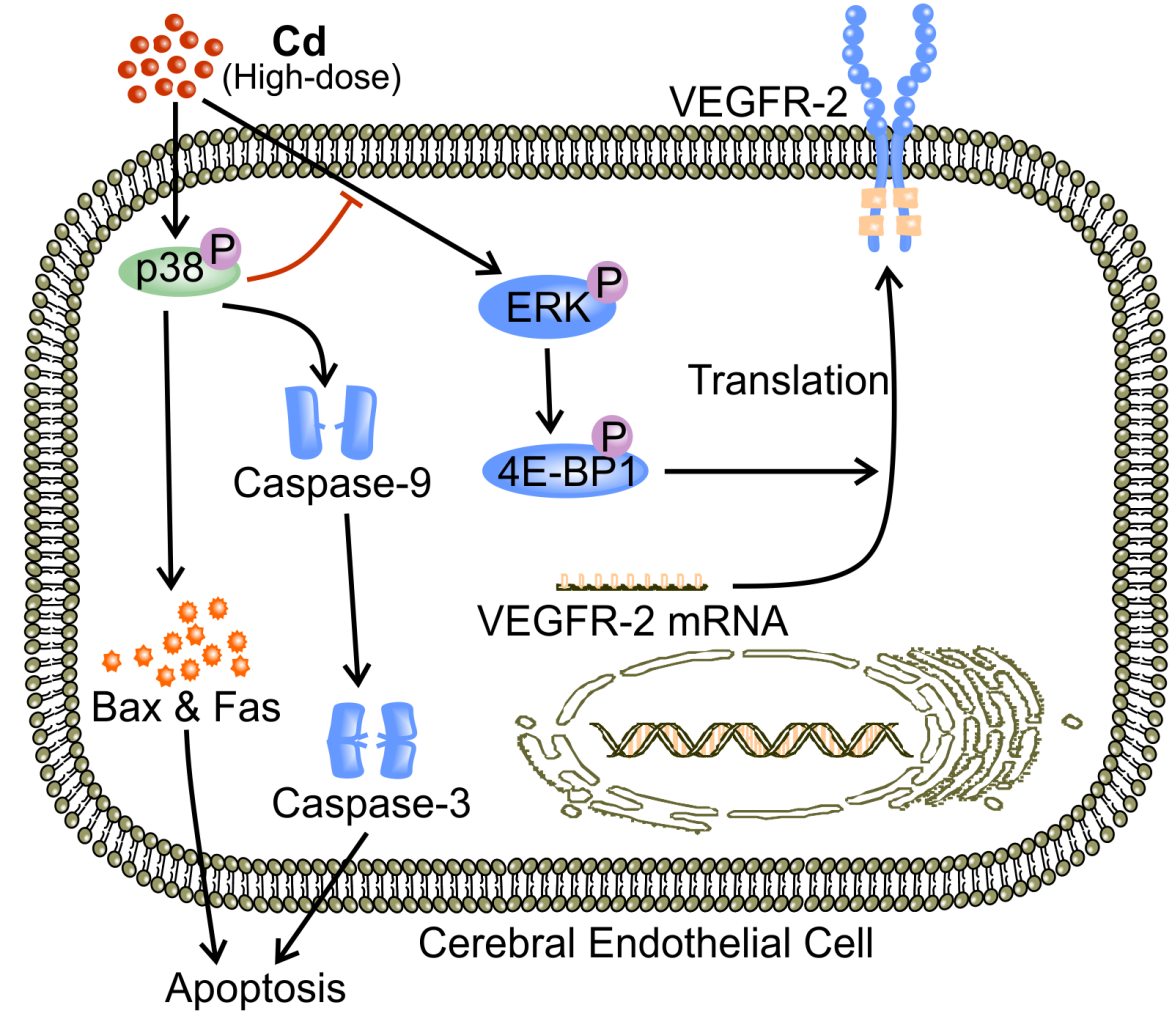
p38 MAPK mediates high-dose Cd induced EC apoptosis At high concentrations ( > 10 μM), Cd triggers EC apoptosis *via* the activation of p38 MAPK [43]. Activation of p38 MAPK increases Bax expression and cleavage of caspase-9 [105, 110, 111]. Caspase-9 cleavage activates caspase-3 [112]. Both increased Bax level and caspase-9-activated caspase-3 are prominent mediators of apoptosis [112]. In addition, phosphorylated p38 MAPK inhibits ERK and impairs the pro-survival VEGF signaling [83, 94, 95, 106, 113].

Consistently through the literature, high dose Cd ( > 10 μM) attenuates angiogenesis *via* the induction of apoptosis [38, 41–43, 107]. As mentioned previously, both p38 MAPK and JNK mediate stress-induced apoptosis [108]. According to Jung et al., Cd (30 μM) activated all three members of the MAPK family in mouse brain microvascular endothelial cells (bEnd.3) while leading to apoptosis [43]. However, only the inhibition of p38 MAPK results in improved survival, suggesting p38 MAPK is the only active MAPK that mediates high-dose Cd-induced EC apoptosis [43]. Indeed, p38 MAPK activation increases the levels of pro-apoptotic proteins including Bax and Fas [105, 109], and is associated with cleavage of caspase-9 and the subsequent activation of caspase-3 [110–112]. p38 MAPK is also postulated to inhibit the pro-survival ERK signaling [16, 106]. Inhibited ERK activation reduces phosphorylated 4E-BP1, leading to less eIF-4E release and eventually lower rates of protein synthesis, which presumably involves decreased VEGFR-2 expression in ECs [83, 113] (Figure 5). Remarkably, with the supposed inhibition of p38 MAPK, ERK activity following Cd-exposure remains elevated [38]. The eventual apoptosis might indicate that the induced level of ERK activation is insufficient for cells to survive under stress of such intensity [38]. As described previously, Cd-stimulated JNK activation, triggering EC apoptosis [67]. Moreover, it appears that the well-known inhibition of JNK by NF-κB is conserved in Cd-treated ECs [67, 81, 82]. Inhibition of JNK activation by NF-κB might explain the failure of JNK inhibitor to preserve cell viability [43]. Future investigation of the role of NF-κB in ECs treated with high-dose Cd may help explain whether phosphorylated JNK is an essential component of Cd-induced apoptosis.

According to Kim et al., phosphorylation of MAPKs in Cd-treated HUVECs were reduced to basal levels when the concentration of Cd exceeded 10 μM but the level of pro-caspase-3 increased with elevation in Cd concentration [38]. Hence, caspase-3 is the major contributor to Cd-related damage in ECs [38, 43]. Although activation of either JNK or p38 seems to be required for apoptosis mediated by caspase-3 [43, 114, 115], the lack of obvious change in levels of these MAPKs suggests that Cd-induced EC apoptosis might involve other activators of caspase-3 [38, 116]. Therefore, regardless of the up-stream signaling cascade involved, high-dose Cd inhibits tumor angiogenesis by inducing caspase-3-mediated apoptosis [38, 43].

With similar Cd concentrations and similar exposure time, Kim et al. observed MAPK activation patterns that were inconsistent with the results of Jung et al. [38, 43]. The only apparent difference between these studies is the variation in EC types [38, 43]. It is well established that ECs with distinct origins exhibit different gene expression patterns, enzymatic activity, and signal transduction [117–119]. In particular, differences in VEGF-induced MAPK activation have long been recognized to depend on the origin of ECs [120]. Treating primary cultures of HUVEC, human aortic EC, and human microvascular EC with the same doses of VEGF for the same time period resulted in differential ERK activation [120]. The cerebral vascular EC used by Jung and colleagues possesses distinct protein expression pattern from peripheral vascular ECs, potentially resulting in disparities in protein activation [48, 121]. Therefore, endothelial heterogeneity may explain the differences in MAPK phosphorylation pattern across these studies.

**Figure 6:**
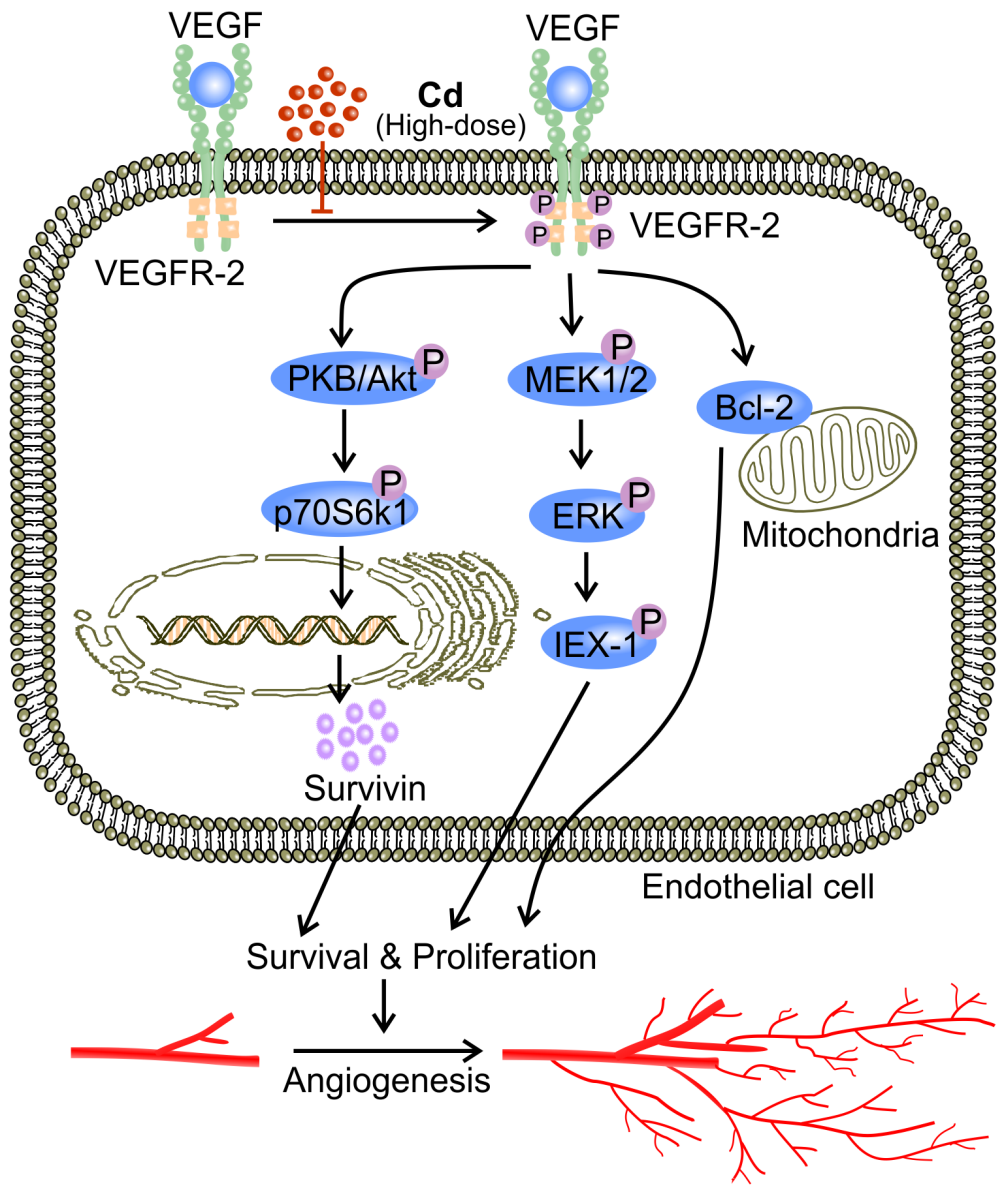
High-dose Cd blocks VEGF signaling and impairs EC survival Phosphorylation of VEGFR-2 TK as a result of binding between VEGF and VEGFR-2 up-regulates inhibitors of apoptosis including Bcl and survivin [42, 72, 138]. VEGF signaling also phosphorylates ERK1/2 *via* MEK1/2, activating IEX-1, an inhibitor of stress-induced apoptosis [70, 113, 124, 125]. High concentrations of Cd ( > 10 μM) block VEGFR-2 activation, and impaired VEGF signaling leads to EC apoptosis and ultimately attenuated angiogenesis [42].

In addition, Cd in high concentrations disrupts signaling pathways that are important to vascular maintenance and growth [3, 41, 42, 122]. A high concentration of Cd significantly decreases both VEGF and VEGFR-2 expression, and thus impairs VEGF signaling [42]. In addition to promoting tumor angiogenesis, VEGF signaling protects against Cd-induced apoptosis through a number of mechanisms [70, 80]. VEGF up-regulates Bcl-2, which promotes survival by inhibiting caspase activation [42]. VEGF also activates Akt to stimulate production of pro-survival proteins such as survivin [12, 72]. Furthermore, VEGF triggers ERK1/2 activation *via* MEK1/2 [70, 113, 123]. ERK activation phosphorylates IEX-1 and inhibits stress-induced apoptosis [124, 125] (Figure 6). In addition, high-dose Cd inhibits the activation of VEGFR-2 tyrosine kinase (TK) activity [126] (Figure 6). Cd might chelate to ATP and form Cd-ATP which could compete with Mg-ATP for enzyme activation sites on VEGFR-2 TKs [126]. With the assumption of Cd-ATP being a slow substrate for TK activation, the accumulation of Cd-ATP could inhibit VEGFR-2 phosphorylation [126]. Alternatively, high dose Cd competes with Mg for the putative second metal-binding site on VEGFR-2 [126]. Therefore, high dose Cd inhibits the activation of VEGFR-2, preventing the down-stream pro-survival signaling transduction [126]. Together, these mechanisms aggravate the cytotoxicity of high-dose Cd while attenuating angiogenesis [42]. Furthermore, high-dose Cd (50 μM) increased the level of angiopoetin-2 (Ang-2) while impairing vascular growth in chick embryos [41]. In the absence of VEGF, high concentrations of Ang-2 inhibit Tie-2 signaling as it displaces Ang-1, the more active ligand, from the receptor [41, 127]. Since Tie-2 signaling is required for both vascular maintenance and response to angiogenic stimuli, impairment of Tie-2 signaling by high-dose Cd might attenuate angiogenesis [3, 41, 122, 128].

## MECHANISM UNDERLYING THE DOSE DEPENDENCY OF CD ACTIONS

The effect of Cd has been characterized as dose dependent both *in vitro* and *in vivo* [31, 129–131]. However, the mechanisms underlying such a property remain elusive. Oxidative stress, characterized by elevated level of ROS, is the primary mediator of Cd toxicity [9, 132, 133], but damage caused by low levels of oxidative stress can be neutralized by anti-oxidant enzymes [134]. After exposure to low-dose Cd, expression and activity of antioxidant enzymes including MT, catalase, glutathione S-transferase, glutathione peroxidase, and quinone oxidoreductase were substantially increased along with the cellular level of GSH [32, 134–136]. Sub-apoptotic levels of oxidative stress also trigger adaptive responses in affected cells [32]. Protection by antioxidant enzymes together with the activation of pro-survival signaling contribute to enhanced cell proliferation [137]. Excessive oxidative stress, however, overwhelms the cellular defense mechanisms and initiates apoptosis to dispose of the damaged cells [137]. Prolonged exposure to increased concentrations of Cd induces a decrease in intracellular GSH level despite the antioxidant enzyme activity, resulting in a significant reduction in cell viability [134]. Therefore, variation in the level of oxidative stress by exposure to Cd of different concentrations potentially explains the dose dependent effect of Cd.

The variations of the results might also be caused by different experimental settings. Here we propose an investigation using HUVEC as the only cell type of interest. The effects on cultured HUVECs are to be evaluated after exposure to different concentrations of Cd representing ultra-low dose, low dose, and high dose. Oxidative stress characterized by intracellular levels of ROS and GSH could be examined to validate our hypothesis that oxidative stress associated with various concentrations of Cd is responsible for the dose-dependent effects of Cd on tumor angiogenesis. Notably, the majority of studies concerning the effect of Cd on angiogenesis have been carried out only *in vitro*. More *in vivo* studies are needed to thoroughly elucidate the effects of different concentrations of Cd on angiogenesis and relevant signaling cascades.

## CONCLUSIONS

As a long-recognized carcinogen, Cd affects tumor angiogenesis at all concentrations [9, 22, 38–43]. The effect of Cd, however, appears to be bi-directional, and is determined by Cd concentration [38]. Ultra-low concentrations of Cd ( < 0.5 μM) inhibit angiogenesis by blocking eNOS activation [39, 40, 49]. Subsequently, reduced level of NO is associated with PHD activation and thus the proteosomal degradation of HIF-1 during hypoxia [61, 65, 66]. The decrease in HIF-1 subsequently reduces VEGF production and angiogenesis [3, 16]. Given that hypoxia is commonly experienced by tumor cells, Cd in ultra-low concentration might in fact attenuate tumor angiogenesis [7, 80]. In contrast, low-dose Cd (1 μM-10 μM) acts on both non-endothelial cells and ECs to promote tumor angiogenesis [9, 35, 67]. Low-dose Cd-induced oxidative stress stimulates the release of VEGF by non-endothelial cells while triggering their malignant transformation [9]. It appears that phosphorylated p70S6K1, resulting from activations of PKB/Akt and ERK, is the primary mediator of this process [9]. p70S6K1 activation increases HIF-1 translation [16, 96]. ERK activation also facilitates the composition of the HIF-1/p300 complex and thus the transcriptional activation of HIF-1 [16]. Activated HIF-1 then binds to the promoter region of VEGF to up-regulate VEGF production [16, 96]. In the mean time, ECs exposed to low-dose Cd demonstrate increased expression of VEGFR-2, which is mediated by activations of NF-κB and all three MAPKs [38, 71]. Low-dose Cd activated NF-κB also up-regulates inhibitors of apoptosis to promote cell survival [67, 81, 82]. Thus, low-dose Cd facilitates tumor angiogenesis by both enhancing VEGF signaling and inhibiting EC apoptosis [9, 38, 67]. Finally, high dose Cd ( > 10 μM) inhibits angiogenesis by triggering EC apoptosis and blocking pro-angiogenic signaling pathways including VEGF and Tie-2 [3, 41, 42]. Exposure to high-dose Cd leads to EC apoptosis mediated by activation of caspase-3 [38, 43, 110, 111], but the relevant upstream signaling event differs across EC types [38, 43]. Moreover, high-dose Cd impairs the ability of non-endothelial cells to produce VEGF while inhibiting the activation of VEGFR-2 in ECs [42, 126]. With reduced VEGF availability, an increase in Ang-2 level following Cd exposure blocks Tie-2 signaling, which is essential for vascular growth [41].

Variations in levels of oxidative stress induced by different concentrations of Cd might explain the dose dependent effect of Cd on tumor angiogenesis. Cells are protected from low levels of oxidative stress owing to antioxidant enzyme activities [134, 137]. Sub-apoptotic levels of oxidative stress trigger adaptive responses, promoting cell survival and proliferation [32]. Excessive oxidative stress induced by high-dose Cd initiates apoptosis of ECs [137]. Mechanisms underlying the effects of Cd on tumor angiogenesis still need to be elucidated. Future investigations using *in vivo* models are needed to further validate current findings.

## Abbreviations

4E-BP1: eukaryotic translation initiation factor 4E-binding protein
Ang-2: angiopoetin-2
AP-1: activator protein-1
BK: bradykinin
CAM: chick chorioallantoic membrane
CaM: camodulin
CaMKII: CaM kinase II
cav-1: caveolin-1
Cd: cadmium
EC: endothelial cell
eIF4B: eukaryotic translation initiation factor 4B
eNOS: endothelial nitric oxide synthase
ERK: extracellular signaling related kinase
FasL: Fas ligand
GSH: glutathione
HEEC: human endometrial endothelial cell
HIF: hypoxia inducible factor
Hsp90: heat shock protein 90
HUVEC: human umbilical vein endothelial cell
IκB: inhibitor κB
JNK: c-Jun N-terminal kinase
MAPK: mitogen-activated protein kinase
MAZ: myc-associated zinc-finger protein
MMP-9: matrix metalloproteinase-9
MNK: MAP kinase interacting kinase
MSK: mitogen- and stress-activated kinases
MT: metallothionein
mTOR: mammalian target of rapamycin
NF-κB: nuclear factor-κB
NO: nitric oxide
O_2_: oxygen
p70S6K: p70S6kinase
PHD: protein hydroxylase domain containing protein
PI3K: phosphatidylinositol-4,5-bisphosphate-3-kinase
PKB: protein kinase B
PKC: protein kinase C
PLC-γ: phospholipase C-γ
ROS: reactive oxygen species
rp: ribosomal protein
Ser: serine
TK: tyrosine kinase
VEGF: vascular endothelial growth factor
VEGFR-2: vascular endothelial growth factor receptor 2
XIAP: X-chromosome linked inhibitor of apoptosis
Zn: zinc
